# Supplementary material for: Prevalence of Chronic Obstructive Pulmonary Disease and Asthma in the Community of Pathumthani, Thailand
Source: Diseases. 2025 Apr 23;13(5):130. doi: 10.3390/diseases13050130 (PMC12109837; doi:10.3390/diseases13050130)
Supplement: Supplementary file 1 [file diseases-13-00130-s001.zip › diseases-3504289-supplementary.pdf]

Table S1. Univariate analysis for factors associated with airway diseases.

Variables	Odds Ratio (95%CI)	*P*-value
Age for every 1-year increase	1.035 (1.021-1.049)	<0.001
Male sex	1.571 (1.123-2.196)	0.008
Body mass index for every 1-unit increase	0.946 (0.930-1.000)	0.049
Smoking	2.286 (1.085-4.956)	<0.001
**Occupations**		
Farmer	1.042 (0.592-1.835)	0.886
Merchant	0.499 (0.320-0.778)	0.002
Unemployed	2.390 (1.732-3.299)	<0.001
**Preexisting comorbidities**		
Hypertension	1.528 (1.102-2.118)	0.011
Coronary heart disease	4.463 (2.254-8.836)	<0.001
Obesity	2.403 (1.000-5.754)	0.055
Allergic rhinitis	1.113 (0.690-1.795)	0.661
**Respiratory symptoms**		
Cough	2.100 (1.456-3.029)	<0.001
Sputum production	1.595 (1.057-2.408)	0.025
Breathlessness	2.917 (1.877-4.534)	<0.001
Wheezes	7.596 (3.101-18.603)	<0.001
Runny nose	2.726 (1.622-4.581)	<0.001
**History of respiratory treatment and cost**		
Previous treatment of dyspnea	8.955 (5.138-15.608)	<0.001
Prior emergency department visit in the past year	2.896 (1.380-6.075)	0.003
